# Making care primary: Medicare's latest attempt at value-based primary care

**DOI:** 10.1093/haschl/qxad072

**Published:** 2023-12-04

**Authors:** Wasan Kumar, Eli Y Adashi, Bob Kocher

**Affiliations:** Stanford University, School of Medicine, Stanford, CA 94305, United States; Brown University, Providence, RI 02913, United States; Stanford Univerisity, Department of Health Policy, USC Schaeffer Center, Venrock, Palo Alto, CA 94304, United States

**Keywords:** value-based payments, risk-based primary care, Centers for Medicare and Medicaid Services (CMS), Centers for Medicare and Medicaid Innovation (CMMI), health care policy, health care economics

## Abstract

On June 8th, 2023, the Centers for Medicare and Medicaid Innovation (CMMI) announced the Making Care Primary (MCP) model, its latest attempt to transform primary care delivery for a value-based care payment system. The MCP is a decade-long multi-payer partnership with a voluntary risk-adjusted payment model for primary care organizations. It provides financial support for organizations to develop and implement a value-based care infrastructure and prospective payments per beneficiary for the delivery of primary care. The MCP consists of 3 tracks, ranging from lump-sum infrastructure payments to a fully prospective payment model with 1-sided risk. In turn, physicians need to meet a set criteria, such as quality outcomes, health-related social needs screening and referral, and high-touch chronic care management (CMMI; https://innovation.cms.gov/innovation-models/making-care-primary). While MCP is a well-planned effort, it is likely to suffer from some of the same pitfalls as prior CMS attempts to revolutionize primary care and may therefore exert unintended effects on market consolidation.

Primary care physicians (PCPs) play a crucial role in preventive care, early detection, and the management of chronic conditions, which can help reduce the need for more expensive specialty care, emergency room visits, and hospitalizations. By focusing on preventive measures and managing health conditions proactively, primary care can prevent costly complications, making it the ideal target for value-based care payment models.

Existing risk-based contracts serve as an example of the potential impact the Making Care Primary (MCP) model can have. Evaluation of the Blue Cross Blue Shield of Massachusetts's Alternative Quality Contract program, a payment model with similarity to MCP, indicated that providers generated 11.7% less spending on claims, with 16.6% fewer lab tests, 21.0% fewer specialty drug prescriptions, and 12.7% fewer emergency department visits.^1^ High-performing Accountable Care Organizations (ACOs) have demonstrated similar results in Medicare.^2^ If similar savings are to be generated via the MCP, we estimate that a PCP could reduce total medical costs by $37 000 in the first year, with the savings per PCP growing to $208 000 over 5 years with a panel of 1000 Medicare patients (Figure 1). The savings generated from lower medical claims will be shared with providers, thereby creating direct incentives to lower the total costs of care. A 2023 report published by Medscape indicated that PCPs, on average, earned $265 000, as compared with their specialist peers who earned, on average, $382 000.^3^ Depending on their capacity for reporting quality metrics and which payment track is chosen, an individual PCP could see their salary from Medicare payments increase by up to 42% over 5 years if they prove successful. Improving profitability of primary care offices will likely lead to beneficial improvements in access to PCPs and improve preventive care.

**Figure 1.**
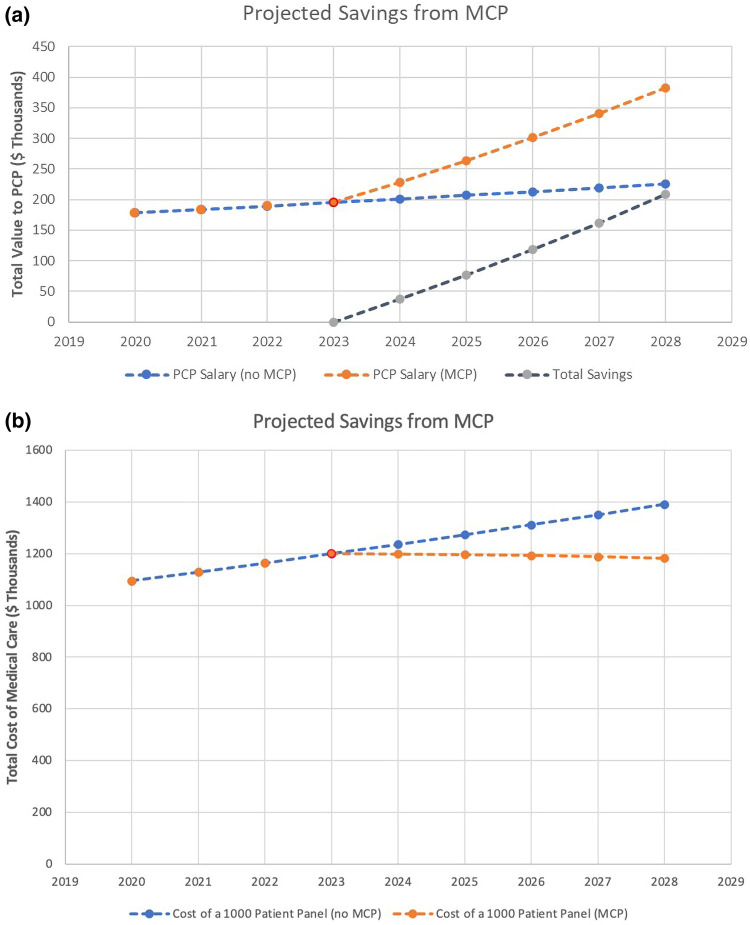
Graph (a): Relationship between medical cost savings and physician income. Estimated value of shared savings for a PCP with a panel of 1000 patients, demonstrating changes in total medical care costs, savings to patients, and PCP salary. Simulated MCP enrollment in 2024, with reference costs of medical care and salary from 2023, is shown. PCP salary adjustment was estimated at 75% of total shared savings. Graph (b): Change in healthcare costs overtime for MCP versus usual care. Total medical care costs were estimated at $1200 per patient, and the standard inflation rate of 3% was assigned. Abbreviations: MCP, Making Care Primary; PCP, primary care provider.

The MCP model is the Centers for Medicare and Medicaid Innovation’s (CMMI's) fifth model for value-based care in the context of primary care. The CMMI tested other primary care models, including the Comprehensive Primary Care (CPC), CPC+, Primary Care First initiatives, and ACO REACH (Realizing Equity, Access, and Community Health). CPC and CPC+ relied on prospective payments to incentivize practices to generate cost savings, yet evaluations of both programs indicated a reduction in hospitalizations with minimal effect on overall Medicare spending.^4,5^ This was, in part, attributed to barriers that clinics faced in developing the infrastructure necessary to implement value-based care initiatives, and a lack of downside risk for clinics, leading to weaker incentives to control costs. The decision not to impute downside risk in the MCP design may again hinder cost control. The Primary Care First and ACO REACH models both aimed to incorporate greater risk-sharing via capitated per-beneficiary payments and performance-based adjustments. The MCP model builds on these experiences via a combination of infrastructure payments and risk-based contracting. MCP plans to enroll participants with no value-based care experience, including Federally Qualified Health Centers, likely spurring innovation in workflow design and health care technology for this space.

Implementing value-based care initiatives will likely require significant capacity building for primary care practices to attain performance-based rewards and achieve quality goals. We envision that many primary care practices may turn to partnering with external organizations for technology, support, and training. This anticipates a challenge that MCP will face, whereby value-based care payments must be more profitable than current fee-for-service reimbursement, net of the startup costs of acquiring technology and training staff, to justify investment and adoption.

Value-based primary care has been a major area of investment and acquisition in 2023, including CVS acquiring Oak Street and Signify Health as well as Humana partnering with Welsh Carson to build CenterWell Medicare Advantage clinics. We anticipate an increase in these purpose-built value-based care practices in the coming years, which may be well suited to fit the MCP model. One risk of success may be that PCP offices become more attractive to private equity buyers since the model increases revenue and profitability of the practices.

Further, the use of risk-adjusted historical coding to determine MCP reimbursement may favor organizations with historically higher risk coding, thereby inadvertently favoring organizations already suited to capturing diagnostic coding revenue. The use of patient social factors, such as area deprivation index, and quality metrics, such as social needs screening, can mitigate this concern but could well lead to the aforementioned difficulties of scaling for clinical operations. Consolidation of primary care has led to concerns for exploiting revenue-capture strategies such as increased intensity of coding, increased price to consumers in fee-for-service models, and less physician autonomy.^6^ Lawmakers could mitigate some of the risks of consolidation by requiring transparency about the ownership of physician practices and pursuing strategies to ensure that acquisitions do not lead to decreased participation in value-based payment programs.

Similar existing initiatives, such as Medicare Advantage and ACOs, with capitated payments and 2-sided risk, have demonstrated that savings can be achieved sustainably by physician practices. This further raises the question of what an MCP program adds, given that there already exists a large menu of federal value-based incentive programs, including ACO REACH, Medicare Advantage, and several tracks of the National Medicare Shared Savings Program. Part of the differentiation may be the performance measures in MCP, which are varied with cost being only one of them, thus incentivizing preventive care in line with what PCP offices are accustomed to, as opposed to the ACO model which more broadly uses shared savings. That said, this approach may decrease the economic incentive for savings and, therefore, the ability for PCPs to justify the investments necessary to redesign workflows to generate savings. A novel aspect of the MCP model is the ability to waive cost-sharing for patients in need, previously prohibited under anti-kickback laws, which may increase access to care for certain patients.

One of the likely outcomes of successful MCP implementation would be a reduction in high-cost specialist referrals, as PCPs are further incentivized to prevent exacerbations of health conditions that would require escalation of care. MCP participants should have a disincentive to refer patients to hospitals due to facility fees and specialists who practice in high-cost contexts as well. This could reduce the overall number of referrals to specialists and redirect some referrals from higher-total-cost to lower-total-cost specialists. Furthermore, specialists remain in a fee-for-service model, creating misaligned incentives for cost control. The MCP model, in turn, mandates engagement of specialists via “specialty care partners” to create collaborative care networks with local specialists, and new billing codes exclusive to those in MCP. To offset declining specialty referrals, this development may accelerate tertiary care systems acquisitions of PCP offices.

Overall, the MCP model has ambitious goals to make value-based care work for more primary care offices. The combination of lump-sum payments to improve infrastructure with multi-payer partnerships to provide 1-sided risk and a value-based payment model seems fitting for a field that already provides significant preventive care and population health management. There are potential downsides to this initiative, including a reduction in hospital admissions and specialist referrals and accelerated acquisitions of PCP offices. The initiative itself may well suffer from a lack of differentiation from previous Centers for Medicare and Medicaid Services models and should thus consider policy adjustments, such as the inclusion of downside risk and ways to mitigate health care consolidation.

## Supplementary Material

qxad072_Supplementary_Data
